# Memory Prosthesis: Is It Time for a Deep Neuromimetic Computing Approach?

**DOI:** 10.3389/fnins.2019.00667

**Published:** 2019-07-04

**Authors:** Vassilis Cutsuridis

**Affiliations:** School of Computer Science, University of Lincoln, Lincoln, United Kingdom

**Keywords:** deep learning, neuromimetic architecture, neuromimetic computing, closed loop stimulation, memory implants

## Abstract

Memory loss, one of the most dreaded afflictions of the human condition, presents considerable burden on the world's health care system and it is recognized as a major challenge in the elderly. There are only a few neuromodulation treatments for memory dysfunctions. Open loop deep brain stimulation is such a treatment for memory improvement, but with limited success and conflicting results. In recent years closed-loop neuroprosthesis systems able to simultaneously record signals during behavioral tasks and generate with the use of internal neural factors the precise timing of stimulation patterns are presented as attractive alternatives and show promise in memory enhancement and restoration. A few such strides have already been made in both animals and humans, but with limited insights into their mechanisms of action. Here, I discuss why a deep neuromimetic computing approach linking multiple levels of description, mimicking the dynamics of brain circuits, interfaced with recording and stimulating electrodes could enhance the performance of current memory prosthesis systems, shed light into the neurobiology of learning and memory and accelerate the progress of memory prosthesis research. I propose what the necessary components (nodes, structure, connectivity, learning rules, and physiological responses) of such a deep neuromimetic model should be and what type of data are required to train/test its performance, so it can be used as a true substitute of damaged brain areas capable of restoring/enhancing their missing memory formation capabilities. Considerations to neural circuit targeting, tissue interfacing, electrode placement/implantation, and multi-network interactions in complex cognition are also provided.

Memory is important in our lives. It is our brain's filing system. Without memory we are unable to remember our past experiences and our loved ones, yet be able to think about the future. Without memory we cannot learn anything. Loss of ability to remember is one of the most dreaded afflictions of the human condition and presents considerable and rising social and economic costs on the world's health and social care systems in the context of the increasing aging of the world's population. Brain disorders such as Alzheimer's disease (AD) and Traumatic Brain Injury (TBI) lead to profound memory deficits and are recognized as major challenges and one of the most important causes of disability in the elderly.

Unfortunately, there are only a few non-pharmacological neuromodulation treatments (Guo et al., 2002; Sjögren et al., 2002; Solé-Padullés et al., 2006; Mannu et al., 2011; Suthana et al., 2012) which alter the course and symptoms of these brain disorders. Direct deep-brain stimulation (DBS) has emerged in the last decade as a neuromodulation technique to treat memory dysfunctions (Hu et al., 2009; Arrieta-Cruz et al., 2010; Laxton et al., 2010; Stone et al., 2011; Boggio et al., 2012; Lyketsos et al., 2012; Suthana et al., 2012; Fell et al., 2013; Hardenacke et al., 2013; Hescham et al., 2013a, 2015; Lee D. J. et al., 2013; Suthana and Fried, 2014; Sweet et al., 2014; Lee et al., 2015; Sankar et al., 2015; Zhang et al., 2015; Jacobs et al., 2016; Lozano et al., 2016; Rezai et al., 2016), but with limited success and contradicting results. A review of all DBS studies is beyond the scope of this article. Interested readers should refer to Bick and Eskandar (2016); Khan et al. (2019); Curot et al. (2017); Ezzyat and Rizzuto (2018) for excellent extensive reviews of the effects of DBS on all memory-related brain areas. Below I briefly review a few of these conflicting studies. In one study DBS at 50Hz applied to human entorhinal cortex (EC) enhanced spatial memory, while hippocampal stimulation did not affect performance (Suthana et al., 2012), whereas in another study DBS at 50 Hz application to both human EC and hippocampus (HC) disrupted spatial and verbal memory (Jacobs et al., 2016). In both studies DBS was applied during the encoding phase, and recall performance was tested when stimulation was off. In another study when 50 Hz DBS was applied between the encoding and recall periods in the left medial temporal lobe (MTL) of patients, then memory recall was impaired (Merkow et al., 2017). Direct electrical stimulation at 50 Hz in HC, parahippocampal regions, prefrontal cortex and lateral temporal cortex (LTC) found that high gamma activity induced by word presentation was decreased in regions where stimulation decreased memory performance, and increased in LTC where memory enhancement was observed (Kucewicz et al., 2018). In other studies, memory impairment was observed when both hippocampi were stimulated simultaneously (Lacruz et al., 2010), but the type of impairment depended on which hippocampus was stimulated (Coleshill et al., 2004). Theta-burst micro-stimulation with physiologic level currents in the right EC during learning significantly improved memory specificity for novel portraits as well as recognition of previously-viewed photos, but not for similar lures (Titiz et al., 2017). On the other hand, theta-burst stimulation of human MTL resulted in spatial memory retrieval impairment (Kim et al., 2018). Theta-burst stimulation in amygdala or fornix (FX) in humans led to visuo-spatial memory enhancement (Miller et al., 2015; Inman et al., 2018). Chronic DBS at 130–450 Hz for several months showed no significant or subtle improvement in memory (Velasco et al., 2007; McLachlan et al., 2010; Boëx et al., 2011; Miatton et al., 2011). Bilateral 20Hz DBS of nucleus basalis of Meynert (NBM) showed memory improvement when stimulation was applied at an earlier stage of dementia and a younger age cohort (Kuhn et al., 2015). Bilateral DBS of anterior thalamic nucleus (ATN) of an epilepsy patient cohort showed greater subjective memory impairment when the stimulation was on and improved word fluency and verbal memory (Fisher et al., 2010; Oh et al., 2012).

Similar conflicting results have been observed in animal studies. Intermittent stimulation in NBM in adult monkeys enhanced working memory, but continuous stimulation led to memory impairment (Liu et al., 2017). EC stimulation in rats promoted neurogenesis in dentate gyrus and enhanced spatial memory in a water maze task in a manner dependent on neurogenesis (Stone et al., 2011). Chronic DBS in Alzheimer's disease (AD) mice improved performance in Morris water maze task with AD-DBS mice spending more time at the novel object and location than with AD-no stimulation mice (Mann et al., 2018). EC, FX, and region CA1 stimulation during a spatial memory study restores performance in a rat scopolamine injection dementia model (Hescham et al., 2013b, 2015), whereas in another study DBS of EC and FX showed significant HC-dependent spatial memory improvement in Morris water maze than in ATN DBS (Zhang et al., 2015). HC-independent recognition memory was also enhanced by EC and FX DBS, but not with ATN DBS (Zhang et al., 2015). Low-current stimulation of rostral intralaminar thalamic nuclei in rats just prior to memory retrieval in a delayed match-to-sample task improved performance, whereas high-current stimulation impaired it (Mair and Hembrook, 2008).

These conflicting results are due to methodological differences across human and animal studies including but not limited to details in participants (age, cognitive, and neurologic abnormalities), animal species (rats, mice, monkeys), behavioral task design, electrode characteristics (e.g., electrode geometry), electrode placements (location), stimulation parameters (amplitude, impedance, frequency, duration, charge density), timing of stimulation (during encoding phase, during retrieval phase, in-between encoding, and retrieval), mode of stimulation (intermittent, chronic, continuous) and statistical analysis methods (Montgomery and He, 2016; Suthana et al., 2018). Open-loop DBS generates only pre-programmed high frequency electrical stimulations without being able to receive feedback from the current brain state. Because of its therapeutic effectiveness, clinical innervations have so far preceded the scientific understanding of its mechanisms of action (McIntyre et al., 2004).

Future advances in memory prosthesis technology should thus address fundamental questions on its therapeutic mechanisms of action. They should also be closed-loop (i.e., receive feedback from the current brain state), capable of online self-adaptation to time-varying environments, and amenable to low-power hardware implementations for memory restoration and rehabilitation (Senova et al., 2018). They should be able to simultaneously record neural signals during behavioral tasks and then with the use of internal factors of the neural state determine the precise timing of stimulation (e.g., stimulating at a particular phase of an ongoing endogenous neural oscillation), or make the decision whether to stimulate at all (Hampson et al., 2013; Deadwyler et al., 2017; Ezzyat et al., 2018). Developments toward the latter direction have already been attempted (Berger et al., 2008, 2011; Deadwyler et al., 2017; Ezzyat et al., 2017, 2018). The Ramp project (Ramp project)^1^ examined the efficacy of a biohybrid architecture of tightly coupled natural and neuromorphic hardware neurons. CoroNet (Coronet FP7 project)^2^ developed the scientific and technological foundations for future “bio-hybrid” devices that will combine biological and artificial nervous tissues. DARPA's RAM project (DARPA RAM project)^3^ aims to develop and test a wireless, fully implantable neural-interface medical device for human clinical use. The Human Brain Project (Human Brain Project)^4^ although not directly contributing in the biohybrid/implant direction, it indirectly contributes to it with its neuromorphic hardware (1Mio cores Spinnaker machine) and brain simulation platform.

The first stride toward a closed-loop implantable memory prosthesis system was conducted by Berger et al. (Song et al., 2009; Berger et al., 2010, 2011; Hampson et al., 2012) as an artificial bridge between the chemically lesioned CA3 and CA1 synaptic connections in a rat's hippocampus, when the animal was trained to perform a delayed non-matched sample (DNMS) task. The chip consisted of three components: (1) a recording multi-electrode array (MEA), (2) a very large scale integration (VLSI) implemented multi-input multi-output (MIMO) prediction model of neural activity based on the recorded neural signals, and (3) a stimulating MEA driven by the MIMO predicted neural activities. The MIMO predicted spiking neural activity was based on five electrophysiological mechanisms: (i) a feedforward process transforming the input MEA recorded spike train to a synaptic potential, (ii) a feedback process generating an after-potential caused by the output spike, (iii) an intrinsic neuronal noise, (iv) a subthreshold potential dynamics, and (v) a threshold function to generate each output spike. When the chip was tested against the damaged CA3-CA1 connection in the lesioned rat, the animal was able to successfully perform the DNMS task with a success rate of over 90% (the success rate for a lesioned rat without the prosthetic device was <50%), demonstrating the chip as a viable memory enhancement device. A second stride toward memory improvement by the chip was made by the same group in non-human primates trained in a delayed match-to-sample (DMS) task (Deadwyler et al., 2017). Despite the chip's successes, it had several limitations. First, it was tested against a single behavioral task on a well-trained animal. That meant the model was “trained” to perform a single input-output mapping. Furthermore, the model was non-adaptive (hard-wired), unable to improve its performance through experience according to a prescribed learning rule. Initial attempts toward the latter direction have been recently made by the same group by incorporating a phenomenological spike timing-dependent plasticity (STDP) rule in an updated MIMO model (Song et al., 2014). However, its synaptic plasticity rule was far too simplistic to capture the complex molecular and biochemical dynamics of synaptic plasticity *in vivo* (Froemke and Dan, 2002; Froemke et al., 2005; Wang et al., 2005). Both MIMO models were completely blind to the CA3 circuit memory computations and processes during their therapeutic courses of action.

A third stride toward a closed-loop memory enhancement/restoration stimulation system was recently made by Ezzyat et al. (2017, 2018) using a machine learning (ML) approach. A set of stimulation-free trials with neural data and labels indicating memory performance was collected from 25 neurosurgical patients undergoing clinical monitoring for epilepsy while they participated in a delayed free recall memory task. A multivariate classifier model was then trained to discriminate patterns of neural activity during encoding for each particular participant. The resulting weight codes from training were then used during testing to map features of iEEG activity to an output probability value, which in turn generated appropriate stimulation patterns during a later word recall phase. Improved memory recall performance was demonstrated particularly when stimulation was timed to periods of poor memory function. Despite its memory improvement success, the closed-loop stimulation system was completely “blind” to the neurobiology of learning and memory offering no insights into the biophysical mechanisms of action of DBS stimulation of the human lateral MTL when participants perform a memory recall task.

With the advent of new and more advanced experimental techniques (Boyden, 2015; Grosenick et al., 2015; Grossman et al., 2017; Kim et al., 2017; Chen et al., 2018; Hardt and Nadel, 2018; Lee and Brecht, 2018), a wealth of knowledge about the anatomical, physiological, molecular, synaptic and connectivity properties of the various cell types in memory-related circuits has accumulated (Cutsuridis et al., 2010a, 2019; Prager et al., 2016; Sprekeler, 2017; Lucas and Clem, 2018). Apart from the numerous different identified classes of interneurons targeting specific parts of excitatory cells (Freund and Buzsáki, 1996; Markram et al., 2004; Klausberger and Somogyi, 2008; Ehrlich et al., 2009; Karnani et al., 2014; Prager et al., 2016; Tremblay et al., 2016; Sprekeler, 2017; Krabbe et al., 2018) and a complex set of intra- and extra-areal excitatory inputs targeting them (Witter, 2019) there is also increasing evidence on the important role of inhibition between interneurons (Chamberland and Topolnik, 2012) in sculpting their activity and entraining them to fire with respect to ongoing network oscillations (Somogyi et al., 2013; Roux and Buzsáki, 2015; Cardin, 2018). Synapses on excitatory and inhibitory cells have been shown to undergo various forms of long-term plasticity (LTP/LTD/STDP, branch potentiation, clustered plasticity, metaplasticity) across different timeframes (ms, seconds, minutes, hours, days, longer) (Govindarajan et al., 2006; Citri and Malenka, 2008; Losonczy et al., 2008; Froemke, 2015; Hattori et al., 2017; Hennequin et al., 2017; Lamsa and Lau, 2019). Hippocampal oriens interneurons display anti-Hebbian long term potentiation, which depends on cholinergic modulation via nicotinic acetylcholine receptors (Griguoli et al., 2013; Rozov et al., 2017). Experimental investigations and compartmental modeling has predicted inhibition of dendritic Ca^2+^ transients modulate the sign and magnitude of synaptic plasticity like long-term potentiation (LTP) or long term depression (LTD) (Cutsuridis, 2011, 2012, 2013; Gidon and Segev, 2012; Jadi et al., 2012; Camiré and Topolnik, 2014) The interaction mechanisms of such molecular, synaptic and cellular components form complex neural circuitries firing at different phases of neuronal oscillations, externally paced or internally generated (Cobb et al., 1995; Buzsaki, 2002; Montgomery et al., 2009), which support different functionalities in health and disease of memory and learning (Marín, 2012; Hangya et al., 2014; Wester and McBain, 2014; Caroni, 2015; Prager et al., 2016; Maffei et al., 2017; Villette and Dutar, 2017; Lucas and Clem, 2018; Vargova et al., 2018). Only by linking this wealth of information into coherent theoretical frameworks (Cutsuridis and Wenneckers, 2009; Cutsuridis et al., 2010b, 2011; Cutsuridis and Hasselmo, 2012; Pendyam et al., 2013; Bezaire et al., 2016) light will be shed into the therapeutic mechanisms of action of any memory enhancement/improvement system. Thus, with the recent exponential increase in computational power, it is thus imperative for the experimental including medical and computational communities to communicate with each other more closely, in order to decipher the molecular, synaptic, cellular, circuit, and systems mechanisms by which closed-loop neuromodulation system operates in memory enhancement, restoration, and rehabilitation and accelerate the progress in memory prosthesis research.

Below, I provide few guidelines on how to construct such a system. I propose that a computational deep (multi-layered) neuromimetic circuit approach empowered with biophysically realistic learning rules mimicking the neural dynamics of memory related circuits amenable to neuromorphic VLSI hardware driven by *in-vivo* MEA recordings, able to decode memory engrams and stimulate memory related populations of neurons should be adopted to move forward the memory prosthesis research. Model components (nodes, synapses, connectivity) should have to mimic the operations of real neurons, synapses and circuits. Several strides toward this direction have already been made (Cutsuridis and Wenneckers, 2009; Cutsuridis et al., 2010b, 2011; Cutsuridis and Hasselmo, 2012; Schneider et al., 2012; Pendyam et al., 2013; Bezaire et al., 2016; Sanjay and Krothapalli, 2019; Yu et al., 2019). One such stride was the Cutsuridis et al. (2010b) microcircuit model of region CA1 dynamics in encoding and retrieval of memories. The study explored the functional roles of somatic, axonic and dendritic inhibition during these processes. It showed how theta modulated inhibition separated encoding and retrieval of memories in the hippocampus into two functionally independent processes. The study predicted: (1) somatic inhibition allowed generation of dendritic calcium spikes that promoted synaptic LTP, while minimizing cell output, (2) proximal dendritic inhibition controlled both cell output and suppressed dendritic calcium spikes, thus preventing LTP, and (3) distal dendritic inhibition removed interference from spurious memories during recall. Some of the Cutsurdis et al. study's predictions have been recently verified by experimental studies (Siegle and Wilson, 2014). The model should also be empowered with biophysically realistic learning rules (LTP/LTD/STDP, branch potentiation, clustered plasticity, metaplasticity, error driven Hebbian learning, etc) mimicking the processes and operations of synaptic plasticity across different timeframes (ms, seconds, minutes, hours, days, longer) in neural cells (Kastellakis et al., 2015, 2016; Li et al., 2016). Once the model's neural dynamics has been extensively validated against experimental data from multiple levels of detail (molecular, synaptic, cellular, dendritic, micro-, meso- and macro-circuit), thus casting it as a faithful representation of a real human/animal tissue (memory circuit), then the model should be trained with real MEA recording and stimulation data from humans or animals while they are performing memory-related behavioral tasks and with verified memory restoration/enhancement effects. Deficits should be in the encoding and/or retrieval of declarative memories (or specific types of declarative memories). Behavioral memory tasks should assess performance metrics across various timeframes (hours, days, weeks, or longer) testing different memory specificities (e.g., memory of an object, event, or context in which it occurs, or high-level semantics of sets of objects/events, or an association of an object and an event linked to one another in a memory occurring either simultaneously or in a temporal sequence). MEA data should be split in training, cross-validation and testing datasets. Model's performance must be tested across individual participants and/or the whole participant population and it must be able to retain functionality across time, situational contexts, and/or experimental settings (tasks). Model robustness and generalization should be validated within and across individual human participant and/or animal and should be demonstrated by the ability of the model to restore memory function when applied to different human participants/animals and in different situational contexts.

Once the model has been computationally trained and its performance have been extensively tested across individuals, experimental settings, memory types and situational contexts, then its structure and weight codes can be transferred to a neuromorphic chip to be implanted or interfaced with indwelling probes for recording and stimulation of human and/or animal neural activity. At this point a number of other outstanding technical difficulties need to be overcome and questions to be answered:

- Electrode Placement and Implantation: The exact placement and trajectory path for the recording and simulation electrodes is of paramount importance to any successful implantable neuroprosthesis system. Any slight deviation from the optimal path to the target due to lead migration or misplacement may result in adverse effects such as hemorrhage, seizures, abnormal sensations, etc or tissue damage (Edwards et al., 2017). Electrode location thus must be adjusted to maximize therapeutic effects, while minimize adverse ones (Edwards et al., 2017). Intra/post-operative imaging (e.g., MRI or CAT) scans can confirm electrode placement (Edwards et al., 2017).
- Neural Circuit Targeting: The electrical field generated by a DBS macroelectrode affects the three-dimensional geometry of the surrounding to the electrode neural processes (i.e., axons and dendrites) (McIntyre et al., 2004). Knowing the anatomical distribution of the DBS electric field and controlling its shape is of utmost importance to maximize the therapeutic effect of stimulation, minimize its adverse effects, and get a deeper understanding of the DBS mechanisms of action (Klooster et al., 2016; Edwards et al., 2017). Electrode design (size, diameter, number of contacts) and directional steering is an active experimental and theoretical research area (Klooster et al., 2016). Mathematical models using finite difference or finite element methods model the electric field induced in the brain during DBS as a function of different stimulation parameters and delineate the effects the electric field has on the neural tissue. The importance of specific conductivities, encapsulation layers and steering toward the stimulation target are some of the main focuses of these studies (Wei and Grill, 2005; Johnson and McIntyre, 2008; Vasques et al., 2009; Schmidt and van Rienen, 2012a,b; Lempka and McIntyre, 2013). Recently developed neural probes have provided precision in shaping the electrical field generated during stimulation (Klooster et al., 2016). One such probe is the “SureSTIM” (Martens et al., 2011), a 64 disc-shaped electrode array arranged in 16 equally-spaced rows, which allows for both long-term stimulation and local field potential recording, while diminishes the induction of adverse effects by stimulating tissue beyond the stimulation target.
- Neural Tissue Interfacing and Longevity: Brain-chip interfaces allow for chips and nerve tissue to establish a close physical interaction thus allowing the transfer of information in one or both directions (Vassanelli et al., 2012). Major operations, like cognition including memory, are sustained by the concurrent activity of a large number of neurons in complex neural networks located in several interconnected brain structures. To better understand neural circuit operations and to develop powerful brain-machine interfaces, then an interface between a semiconductor chip or an ensemble of chips and the neural tissue of a living animal allowing for bi-directional communication (not only to record but also to control neuronal activity) and high-spatiotemporal resolution sampling of a large number of neurons over the networks, and simultaneously from multiple regions of the brain is needed (Vassanelli et al., 2012). Usually small CMOS chips featuring stimulation and recording sites integrated at high-density implanted in one or in several brain areas, either independently or simultaneously, can lead to an unprecedented control of neuronal activity in the mammalian brain (CyberRat ICT 2007 project)^5^ Obtaining such high spatiotemporal resolution enables to explore and control brain information processing with unprecedented detail. The chips are either directly implanted into the tissue or connected through leads that reside permanently in the brain. Wireless transmission is desired to simplify chips connectivity with the monitoring system and to remove interference with animals' movements (Vassanelli et al., 2012). Several bottlenecks are usually faced: power dissipation induced heat generation of the chips, biocompatibility and mechanical-electrical stability, particularly for chronic implantation in the freely behaving animal, chip implantation (and chip design) to match at best the 2D architecture of the array with the 3D architecture of the neuronal networks in the brain while limiting to the minimum tissue damage (Vassanelli, 2018).
- Multi-Network Interactions in Complex Cognition: For a long time, it was hypothesized that DBS worked either via functional ablation by suppressing or inhibiting the structure being stimulated or via activation of the stimulated structure (McIntyre et al., 2004). It is currently accepted that DBS changes network-wide oscillations and there may be coherence between cortical and subcortical brain signals (Wagle Shukla and Okun, 2012; Lee H. et al., 2013). Are these changes though due to a widespread DBS electric field affecting circuits/areas/regions well beyond the stimulated one (global effects) or due to a localized electric field affecting only the DBS brain circuit/region/area, which in turn drives other connected with it brain circuits/regions/areas (local effects)? A notable study on uncovering the mechanisms of whole-brain dynamics of deep brain stimulation has shown that DBS shifts global brain dynamics of patients toward a healthy regime with the effect more pronounced in specific brain areas (Saenger et al., 2017). Higher communicability and coherence in brain areas were measured when DBS was on than then it was off (Saenger et al., 2017).

Overall, to accelerate progress in memory prosthesis technologies then a closed-loop deep neuromimetic circuit computing approach empowered with biophysically realistic learning rules mimicking the neural dynamics of memory related circuits amenable to neuromorphic VLSI hardware driven by *in-vivo* MEA recordings, able to decode memory engrams and stimulate memory related populations of neurons should be adopted. Such software novelties along with multimodal neuroimaging, electrophysiological and electrochemical monitoring technologies and innovative neural probe engineering advances (e.g., SureSTIM) could then act as true substitutes (bridges) of damaged memory-related brain areas capable of restoring/enhancing their missing memory formation capabilities as well as deciphering their mechanisms of action.

## Data Availability

No datasets were generated or analyzed for this study.

## Author Contributions

The author confirms being the sole contributor of this work and has approved it for publication.

### Conflict of Interest Statement

The author declares that the research was conducted in the absence of any commercial or financial relationships that could be construed as a potential conflict of interest.

## Footnotes

^1^Ramp project. Available online at: http://www.rampproject.eu

^2^Coronet FP7 Project. Available online at: http://www.coronet-project.eu

^3^DARPA's RAM project. Available online at: http://www.darpa.mil/program/restoring-active-memory

^4^Human Brain Project. Available online at: https://www.humanbrainproject.eu/en/

^5^CyberRat ICT 2007 project. Available online at: https://www.vassanellilab.eu/projects/cyberrat/

## References

[B1] Arrieta-Cruz I. Pavlides C. Pasinetti G. M. (2010). Deep brain stimulation in midline thalamic region facilitates synaptic transmission and short term memory in a mouse model of Alzheimer's disease. Transl. Neuroscil. 1, 188–194. 10.2478/v10134-010-0023-x 23227306 PMC3515070

[B2] Berger T. W. Gerhardt G. Liker M. A. Sousou W. (2008). The impact of neurotechnology in rehabilitation. IEEE Rev. Biomed. Eng. 1, 157–197. 10.1109/RBME.2008.2008687 22274903

[B3] Berger T. W. Hampson R. E. Song D. Goonawardena A. Marmarelis V. Z. Deadwyler S. A. (2011). A cortical neural prosthesis for restoring and enhancing memory. J. Neural Eng. 8:046017. 10.1088/1741-2560/8/4/046017 21677369 PMC3141091

[B4] Berger T. W. Song D. Chan R. H. Marmarelis V. Z. (2010). The neurobiological basis of cognition: Identification by multi-input, multioutput nonlinear dynamic modelling. Proc. IEEE Inst. Electr. Electron. Eng. 98, 356–374. 10.1109/JPROC.2009.2038804 20700470 PMC2917774

[B5] Bezaire M. J. Raikov I. Burk K. Vyas D. Soltesz I. (2016). Interneuronal mechanisms of hippocampal theta oscillations in a full-scale model of the rodent CA1 circuit. Elife. 5:e18566. 10.7554/eLife.18566 28009257 PMC5313080

[B6] Bick S. K. Eskandar E. N. (2016). Neuromodulation for restoring memory. Neurosurg. Focus 40:E5. 10.3171/2016.3.FOCUS162 27132526

[B7] Boëx C. Seeck M. Vulliémoz S. Rossetti A. O. Staedler C. Spinelli L.. (2011). Chronic deep brain stimulation in mesial temporal lobe epilepsy. Seizure 20, 485–490. 10.1016/j.seizure.2011.03.001 21489828

[B8] Boggio P. S. Ferrucci R. Mameli F. Martins D. Martins O. Vergari M.. (2012). Prolonged visual memory enhancement after direct current stimulation in Alzheimer's disease. Brain Stimulat. 5, 223–230. 10.1016/j.brs.2011.06.006 21840288

[B9] Boyden E. S. (2015). Optogenetics and the future of neuroscience. Nat. Neurosci. 18, 1200–1201. 10.1038/nn.4094 26308980

[B10] Buzsaki G. (2002). Theta oscillations in the hippocampus. Neuron 33, 325–340. 10.1016/S0896-6273(02)00586-X 11832222

[B11] Camiré O. Topolnik L. (2014). Dendritic calcium nonlinearities switch the direction of synaptic plasticity in fast-spiking interneurons. J. Neurosci. 34, 3864–3877. 10.1523/JNEUROSCI.2253-13.2014 24623765 PMC6705275

[B12] Cardin J. A. (2018). Inhibitory interneurons regulate temporal precision and correlations in cortical circuits. TINS 41, 689–700. 10.1016/j.tins.2018.07.015 30274604 PMC6173199

[B13] Caroni P. (2015). Inhibitory microcircuit modules in hippocampal learning. Curr. Opin. Neurobiol. 35, 66–73. 10.1016/j.conb.2015.06.010 26176433

[B14] Chamberland S. Topolnik L. (2012). Inhibitory control of hippocampal inhibitory neurons. Front Neurosci. 6:165. 10.3389/fnins.2012.00165 23162426 PMC3496901

[B15] Chen S. Weitemier A. Z. Zeng X. He L. Wang X. Tao Y.. (2018). Near-infrared deep brain stimulation via upconversion nanoparticle-mediated optogenetics. Science 359, 679–684. 10.1126/science.aaq1144 29439241

[B16] Citri A. Malenka R. C. (2008). Synaptic plasticity: multiple forms, functions, and mechanisms. Neuropsychopharmacology 33, 18–41. 10.1038/sj.npp.1301559 17728696

[B17] Cobb S. R. Buhl E. H. Halasy K. Paulsen O. Somogyi P. (1995). Synchronization of neuronal activity in hippocampus by individual GABAergic interneurons. Nature 378, 75–78. 10.1038/378075a0 7477292

[B18] Coleshill S. G. Binnie C. D. Morris R. G. Alarcón G. van Emde Boas W. Velis D. N.. (2004). Material-specific recognition memory deficits elicited by unilateral hippocampal electrical stimulation. J. Neurosci. 24, 1612–1616. 10.1523/JNEUROSCI.4352-03.2004 14973245 PMC6730466

[B19] Curot J. Busigny T. Valton L. Denuelle M. Vignal J. P. Maillard L.. (2017). Memory scrutinized through electrical brain stimulation: a review of 80 years of experiential phenomena. Neurosci. Biobehav. Rev. 78. 161–177. 10.1016/j.neubiorev.2017.04.018 28445741

[B20] Cutsuridis V. (2011). GABA inhibition modulates NMDA-R mediated spike timing dependent plasticity (STDP) in a biophysical model. Neural Netw. [[Inline Image]] 24, 29–42. 10.1016/j.neunet.2010.08.005 20832991

[B21] Cutsuridis V. (2012). Bursts shape the NMDA-R mediated spike timing dependent plasticity curve: role of burst interspike interval and GABA inhibition. Cogn. Neurodynamics 6, 421–441. 10.1007/s11571-012-9205-1 PMC3438326 24082963

[B22] Cutsuridis V. (2013). Interaction of inhibition and triplets of excitatory spikes modulates the NMDA-R mediated synaptic plasticity in a computational model of spike timing dependent plasticity. Hippocampus 23, 75–86. 10.1002/hipo.22057 22851353

[B23] Cutsuridis V. Cobb S. Graham B. P. (2010b). Encoding and retrieval in the hippocampal CA1 microcircuit model. Hippocampus 20, 423–446. 10.1002/hipo.20661 19489002

[B24] Cutsuridis V. Graham B. P. Cobb S. Vida I. (2010a). Hippocampal Microcircuits: A Computational Modeler's Resource Book, 1st edn. New York City, NY: Springer 10.1007/978-1-4419-0996-1

[B25] Cutsuridis V. Graham B. P. Cobb S. Vida I. (2019). Hippocampal Microcircuits: A Computational Modeler's Resource Book, 2nd edn. Cham: Springer 10.1007/978-3-319-99103-0

[B26] Cutsuridis V. Grahan B. P. Cobb S. Hasselmo M. E. (2011). Bio-inspired models of memory capacity, recall performance and theta phase precession, in Proceedings of the IJCNN (San Jose, CA: IEEE), 141–148. 10.1109/IJCNN.2011.6033637

[B27] Cutsuridis V. Hasselmo M. (2012). GABAergic modulation of gating, timing and theta phase precession of hippocampal neuronal activity during theta oscillations. Hippocampus 22, 1597–1621. 10.1002/hipo.21002 22252986

[B28] Cutsuridis V. Wenneckers T. (2009). Hippocampus, microcircuits and associative memory. Neural Netw. 22, 1120–1128. 10.1016/j.neunet.2009.07.009 19647982

[B29] Deadwyler S. A. Hampson R. E. Song D. Opris I. Gerhrdt G. A. Marmarelis V. Z. (2017). A cognitive prosthesis for memory facilitation by closed-loop function ensemble stimulation of hippocampal neurons in primate brain. Exp. Neurol. 287(Pt 4): 452–460. 10.1016/j.expneurol.2016.05.031 27233622 PMC5633045

[B30] Edwards C. A. Kouzani A. Lee k. H. Ross E. K. (2017). Neurostimulation devices for the treatment of neurologic devices. Mayo Clin. Proc. 92, 1427–1444. 10.1016/j.mayocp.2017.05.005 28870357

[B31] Ehrlich I. Humeau Y. Grenier F. Ciocchi S. Herry C. Lüthi A. (2009). Amygdala inhibitory circuits and the control of fear memory. Neuron 62, 757–771. 10.1016/j.neuron.2009.05.026 19555645

[B32] Ezzyat Y. Kragel J. E. Burke J. F. Gorniak R. Rizzuto D. S. Kahana M. J. (2017). Direction brain stimulation modulates encoding states and memory performance. Curr. Biol. 27, 1251–1258. 10.1016/j.cub.2017.03.028 28434860 PMC8506915

[B33] Ezzyat Y. Rizzuto D. S. (2018). Direct brain stimulation during episodic memory. Curr. Opin. Biomed. Eng. 8, 78–83. 10.1016/j.cobme.2018.11.004

[B34] Ezzyat Y. Wanda P. A. Levy D. F. Kadel A. Aka A. Pedisich I.. (2018). Closed-loop stimulation of temporal cortex rescues functional networks and improves memory. Nat. Commun. 9:365. 10.1038/s41467-017-02753-0 29410414 PMC5802791

[B35] Fell J. Staresina B. P. Do Lam A. T. Widman G. Helmstaedter C. Elger C. E.. (2013). Memory modulation by weak synchronous deep brain stimulation: a pilot study. Brain Stimulat. 6, 270–273. 10.1016/j.brs.2012.08.001 22939277

[B36] Fisher R. Salanova V. Witt T. Worth R. Henry T. Gross R.. (2010). Electrical stimulation of the anterior nucleus of thalamus for treatment of refractory epilepsy. Epilepsia 51, 899–908. 10.1111/j.1528-1167.2010.02536.x 20331461

[B37] Freund T. F. Buzsáki G. (1996). Interneurons of the hippocampus. Hippocampus 6, 347–470. 8915675 10.1002/(SICI)1098-1063(1996)6:4<347::AID-HIPO1>3.0.CO;2-I

[B38] Froemke R. C. (2015). Plasticity of cortical excitatory-inhibitory balance. Annu Rev Neurosci. 38, 195–219. 10.1146/annurev-neuro-071714-034002 25897875 PMC4652600

[B39] Froemke R. C. Dan Y. (2002). Spike timing-dependent synaptic modification induced by natural spike trains. Nature 416:4330438. 10.1038/416433a 11919633

[B40] Froemke R. C. Poo M. M. Dan Y. (2005). Spike timing-dependent synaptic plasticity depends on dendritic location. Nature 434, 221–225. 10.1038/nature03366 15759002

[B41] Gidon A. Segev I. (2012). Principles governing the operation of synaptic inhibition in dendrites. Neuron 75, 330–341. 10.1016/j.neuron.2012.05.015 22841317

[B42] Govindarajan A. Kelleher R. J. Tonegawa S. (2006). A clustered plasticity model of long-term memory engrams. Nat. Rev. Neurosci. 7, 575–583. 10.1038/nrn1937 16791146

[B43] Griguoli M. Cellot G. Cherubini E. (2013). In hippocampal oriens interneurons anti-Hebbian long-term potentiation requires cholinergic signaling via α7 nicotinic acetylcholine receptors. J. Neurosci. 33, 1044–1049. 10.1523/JNEUROSCI.1070-12.2013 23325242 PMC6704869

[B44] Grosenick L. Marshel J. H. Deisseroth K. (2015). Closed-loop and activity-guided optogenetic control. Neuron 86, 106–139. 10.1016/j.neuron.2015.03.034 25856490 PMC4775736

[B45] Grossman N. Bono D. Dedic N. Kodandaramaiah S. B. Rudenko A. Suk H. J.. (2017). Noninvasive deep brain stimulation via temporally interfering electric fields. Cell 169, 1029–1041.e16. 10.1016/j.cell.2017.05.024 28575667 PMC5520675

[B46] Guo Y. Shi X. Uchiyama H. Hasegawa A. Nakagawa Y. Tanaka M.. (2002). A study on the rehabilitation of cognitive function and short-term memory in patients with Alzheimer's disease using transcutaneous electrical nerve stimulation. Front. Med. Biol. Eng. 11, 237–247. 10.1163/156855701321138905 12735425

[B47] Hampson R. E. Song D. Chan R. H. Sweatt A. J. Riley M. R. Gerhardt G. A.. (2012). A nonlinear model for hippocampal cognitive prosthesis: memory facilitation by hippocampal ensemble stimulation. IEEE Trans. Neural. Syst. Rehabil. Eng. 20, 184–197. 10.1109/TNSRE.2012.2189163 22438334 PMC3397311

[B48] Hampson R. E. Song D. Opris I. Santos L. M. Shin D. C. Gerhardt G. A.. (2013). Facilitation of memory encoding in primate hippocampus by a neuroprosthesis that promotes task specific neural firing. J. Neural Eng. 10:066013. 10.1088/1741-2560/10/6/066013 24216292 PMC3919468

[B49] Hangya B. Pi H. J. Kvitsiani D. Ranade S. P. Kepecs A. (2014). From circuit motifs to computations: mapping the behavioral repertoire of cortical interneurons. Curr. Opin. Neurobiol. 26, 117–124. 10.1016/j.conb.2014.01.007 24508565 PMC4090079

[B50] Hardenacke K. Kuhn J. Lenartz D. Maarouf M. Mai J. K. Bartsch C.. (2013). Stimulate or degenerate: deep brain stimulation of the nucleus basalis Meynert in Alzheimer dementia. World Neurosurg 80, S27.e35–S27.e43. 10.1016/j.wneu.2012.12.005 23246738

[B51] Hardt O. Nadel L. (2018). Systems consolidation revisited, but not revised: The promise and limits of optogenetics in the study of memory. Neurosci Lett. 680, 54–59. 10.1016/j.neulet.2017.11.062 29203208

[B52] Hattori R. Kuchibhotla K. V. Froemke R. C. Komiyama T. (2017). Functions and dysfunctions of neocortical inhibitory neuron subtypes. Nat. Neurosci. 20, 1199–1208. 10.1038/nn.4619 28849791 PMC7082034

[B53] Hennequin G. Agnes E. J. Vogels T. P. (2017). Inhibitory plasticity: balance, control, and co-dependence. Annu. Rev. Neurosci. 40, 557–579. 10.1146/annurev-neuro-072116-031005 28598717

[B54] Hescham S. Jahanshahi A. Meriaux C. Lim L. W. Blokland A. Temel Y. (2015). Behavioral effects of deep brain stimulation of different areas of the Papez circuit on memory- and anxiety related functions. Behav. Brain Res. 292, 353–360. 10.1016/j.bbr.2015.06.032 26119240

[B55] Hescham S. Lim L. W. Jahanshahi A. Blokland A. Temel Y. (2013a). Deep brain stimulation in dementia-related disorders. Neurosci. Biobehav. Rev. 37, 2666–2675. 10.1016/j.neubiorev.2013.09.002 24060532

[B56] Hescham S. Lim L. W. Jahanshahi A. Steinbusch H. W. Prickaerts J. Blokland A.. (2013b). Deep brain stimulation of the forniceal area enhances memory functions in experimental dementia: the role of stimulation parameters. Brain Stimulat. 6, 72–77. 10.1016/j.brs.2012.01.008 22405739

[B57] Hu R. Eskandar E. Williams Z. (2009). Role of deep brain stimulation in modulating memory formation and recall. Neurosurg Focus 27:E3. 10.3171/2009.4.FOCUS0975 19569891 PMC2848994

[B58] Inman C. S. Manns J. R. Bijanki K. R. Bass D. I. Hamann S. Drane D. L.. (2018). Direct electrical stimulation of the amygdala enhances declarative memory in humans. PNAS 115, 98–103. 10.1073/pnas.1714058114 29255054 PMC5776809

[B59] Jacobs J. Miller J. Lee S. A. Coffey T. Watrous A. J. Sperling M. R.. (2016). Direct electrical stimulation of the human entorhinal region and hippocampus impairs memory. Neuron 92, 983–990. 10.1016/j.neuron.2016.10.062 27930911

[B60] Jadi M. Polsky A. Schiller J. Mel B. W. (2012). Location-dependent effects of inhibition on local spiking in pyramidal neuron dendrites. PLoS Comput. Biol. 8:e1002550. 10.1371/journal.pcbi.1002550 22719240 PMC3375251

[B61] Johnson M. D. McIntyre C. C. (2008). Quantifying the neural elements activated and inhibited by globus pallidus deep brain stimulation. J. Neurophysiol. 100, 2549–2563. 10.1152/jn.90372.2008 18768645 PMC2585404

[B62] Karnani M. M. Agetsuma M. Yuste R. (2014). A blanket of inhibition: functional inferences from dense inhibitory connectivity. Curr. Opin. Neurobiol. 26, 96–102. 10.1016/j.conb.2013.12.015 24440415 PMC4024353

[B63] Kastellakis G. Cai D. J. Mednick S. C. Silva A. J. Poirazi P. (2015). Synaptic clustering within dendrites: an emerging theory of memory formation. Prog. Neurobiol. 126, 19–35. 10.1016/j.pneurobio.2014.12.002 25576663 PMC4361279

[B64] Kastellakis G. Silva A. J. Poirazi P. (2016). Linking memories across time via neuronal and dendritic overlaps in model neurons with active dendrites. Cell Rep. 17, 1491–1504. 10.1016/j.celrep.2016.10.015 27806290 PMC5149530

[B65] Khan I. S. D'Agostino E. N. Calnan D. R. Lee J. E. Aronson J. P. (2019). Deep brain stimulation for memory modulation: a new frontier. World Neurosurg. 126, 638–646. 10.1016/j.wneu.2018.12.184 30654156

[B66] Kim C. K. Adhikari A. Deisseroth K. (2017). Integration of optogenetics with complementary methodologies in systems neuroscience. Nat. Rev. Neurosci. 18, 222–235. 10.1038/nrn.2017.15 28303019 PMC5708544

[B67] Kim K. Schedlbauer A. Rollo M. Karunakaran S. Ekstrom A. D. Tandon N. (2018). Network-based brain stimulation selectively impairs spatial retrieval. Brain Stimul. 11, 213–221. 10.1016/j.brs.2017.09.016 29042188 PMC5729089

[B68] Klausberger T. Somogyi P. (2008). Neuronal diversity and temporal dynamics: the unity of hippocampal circuit operations. Science 321, 53–57. 10.1126/science.1149381 18599766 PMC4487503

[B69] Klooster D. C. de Louw A. J. Aldenkamp A. P. Besseling R. M. Mestrom R. M. Carrette S.. (2016). Technical aspects of neurostimulation: focus on equipment, electric field modeling, and stimulation protocols. Neurosci. Biobehav. Rev. 65, 113–141. 10.1016/j.neubiorev.2016.02.016 27021215

[B70] Krabbe S. Gründemann J. Lüthi A. (2018). Amygdala inhibitory circuits regulate associative fear conditioning. Biol. Psychiatry 83, 800–809. 10.1016/j.biopsych.2017.10.006 29174478

[B71] Kucewicz M. T. Berry B. M. Kremen V. Miller L. R. Khadjevand F. Ezzyat Y.. (2018). Electrical stimulation modulates high γ activity and human memory performance. eNeuro 5:eNEURO.0369–17. 10.1523/ENEURO.0369-17.2018 29404403 PMC5797477

[B72] Kuhn J. Hardenacke K. Lenartz D. Gruendler T. Ullsperger M. Bartsch C.. (2015). Deep brain stimulation of the nucleus basalis of Meynert in Alzheimer's dementia. Mol. Psychiatry 20, 353–360. 10.1038/mp.2014.32 24798585

[B73] Lacruz M. E. Valentín A. Seoane J. J. Morris R. G. Selway R. P. Alarcón G. (2010). Single pulse electrical stimulation of the hippocampus is sufficient to impair human episodic memory. Neuroscience 170, 623–632. 10.1016/j.neuroscience.2010.06.042 20643192

[B74] Lamsa K. Lau P. (2019). Long-term plasticity of hippocampal interneurons during in vivo memory processes. Curr. Opin. Neurobiol. 54, 20–27. 10.1016/j.conb.2018.08.006 30195105

[B75] Laxton A. W. Tang-Wai D. F. McAndrews M. P. Zumsteg D. Wennberg R. Keren R.. (2010). A phase I trial of deep brain stimulation of memory circuits in Alzheimer's disease. Ann. Neurol. 68, 521–534. 10.1002/ana.22089 20687206

[B76] Lee A. K. Brecht M. (2018). Elucidating neuronal mechanisms using intracellular recordings during behavior. TINS 41, 385–403. 10.1016/j.tins.2018.03.014 29685404

[B77] Lee D. J. Gurkoff G. G. Izadi A. Berman R. F. Ekstrom A. D. Muizelaar J. P.. (2013). Medial septal nucleus theta frequency deep brain stimulation improves spatial working memory after traumatic brain injury. J. Neurotrauma. 30, 131–139. 10.1089/neu.2012.2646 23016534

[B78] Lee D. J. Gurkoff G. G. Izadi A. Seidl S. E. Echeverri A. Melnik M.. (2015). Septohippocampal neuromodulation improves cognition after traumatic brain injury. J. Neurotrauma 32, 1822–1832. 10.1089/neu.2014.3744 26096267 PMC4702430

[B79] Lee H. Fell J. Axmacher N. (2013). Electrical engram: how deep brain stimulation affects memory. TINS 17, 574–584. 10.1016/j.tics.2013.09.002 24126128

[B80] Lempka S. F. McIntyre C. C. (2013). Theoretical analysis of the local field potential in deep brain stimulation applications. PLoS ONE 8:e59839. 10.1371/journal.pone.0059839 23555799 PMC3610913

[B81] Li Y. Kulvicius T. Tetzlaff C. (2016). Induction and consolidation of calcium-based homo- and heterosynaptic potentiation and depression. PLoS ONE 11:e0161679. 10.1371/journal.pone.0161679 27560350 PMC4999190

[B82] Liu R. Crawford J. Callahan P. M. Terry A. V. Jr. Constantinidis C. (2017). Intermittent stimulation of the nucleus basalis of Meynert improves working memory in adult monkeys. Curr. Biol. 27.−2646.e2644. 10.1016/j.cub.2017.07.021 28823679 PMC5759307

[B83] Losonczy A. Makara J. K. Magee J. C. (2008). Compartmentalized dendritic plasticity and input feature storage in neurons. Nature 452, 436–441 10.1038/nature06725 18368112

[B84] Lozano A. M. Fosdick L. Chakravarty M. M. Leoutsakos J. M. Munro C. Oh E.. (2016). A phase II Study of fornix deep brain stimulation in mild Alzheimer's Disease. J. Alzheimers Dis. J. A. D. 54, 777–787. 10.3233/JAD-160017 27567810 PMC5026133

[B85] Lucas E. K. Clem R. L. (2018). GABAergic interneurons: the orchestra or the conductor in fear learning and memory? Brain Res. Bull. 141, 13–19. 10.1016/j.brainresbull.2017.11.016 29197563 PMC6178932

[B86] Lyketsos C. G. Targum S. D. Pendergrass J. C. Lozano A. M. (2012). Deep brain stimulation: a novel strategy for treating Alzheimer's disease. Innov. Clin. Neurosci. 9, 10–17. 23346514 PMC3552463

[B87] Maffei A. Charrier C. Caiati M. D. Barberis A. Mahadevan V. Woodin M. A.. (2017). Emerging mechanisms underlying dynamics of GABAergic synapses. J. Neurosci. 37, 10792–10799. 10.1523/JNEUROSCI.1824-17.2017 29118207 PMC5678011

[B88] Mair R. G. Hembrook J. R. (2008). Memory enhancement with event related stimulation of the rostral intralaminar thalamic nuclei. J. Neurosci. 28, 14293–14300. 10.1523/JNEUROSCI.3301-08.2008 19109510 PMC2630113

[B89] Mann A. Gondard E. Tampellini D. Milsted J. A. T. Marillac D. Hamani C.. (2018). Chronic deep brain stimulation in an Alzheimer's disease mouse model enhances memory and reduces pathological hallmarks. Brain Stimul. 11, 435–444. 10.1016/j.brs.2017.11.012 29246746

[B90] Mannu P. Rinaldi S. Fontani V. Castagna A. (2011). Radio electric asymmetric brain stimulation in the treatment of behavioural and psychiatric symptoms in Alzheimer disease. Clin. Interv. Aging 6, 207–211. 10.2147/CIA.S23394 21822377 PMC3147052

[B91] Marín O. (2012). Interneuron dysfunction in psychiatric disorders. Nat. Rev. Neurosci. 13, 107–120. 10.1038/nrn3155 22251963

[B92] Markram H. Toledo-Rodriguez M. Wang Y. Gupta A. Silberberg G. Wu C. (2004). Interneurons of the neocortical inhibitory system. Nat. Rev. Neurosci. 5, 793–807. 10.1038/nrn1519 15378039

[B93] Martens H. C. F. Toader E. Decré M. M. J. Anderson D. J. Vetter R. Kipke D. R.. (2011). Spatial steering of deep brain stimulation volumes using a novel lead design. Clin. Neurophysiol. 122,558–566. 10.1016/j.clinph.2010.07.026 20729143

[B94] McIntyre C. C. Savasta M. Walter B. L. Vitek J. L. (2004). How does deep brain stimulation work? Present understanding and future questions. J. Clin. Neurophysiol. 21, 40–50. 10.1097/00004691-200401000-00006 15097293

[B95] McLachlan R. S. Pigott S. Tellez-Zenteno J. F. Wiebe S. Parrent A. (2010). Bilateral hippocampal stimulation for intractable temporal lobe epilepsy: impact on seizures and memory. Epilepsia 51, 304–307. 10.1111/j.1528-1167.2009.02332.x 19817814

[B96] Merkow M. B. Burke J. F. Ramayya A. G. Sharan A. D. Sperling M. R. Kahana M. J. (2017). Stimulation of the human medial temporal lobe between learning and recall selectively enhances forgetting. Brain Stimulat. 10, 645–650. 10.1016/j.brs.2016.12.011 28073638 PMC5410394

[B97] Miatton M. Van Roost D. Thiery E. Carrette E. Van Dycke A. Vonck K. (2011). The cognitive effects of amygdalo-hippocampal deep brain stimulation in patients with temporal lobe epilepsy. Epilepsy Behav. 22, 759–764. 10.1016/j.yebeh.2011.09.016 22030536

[B98] Miller J. P. Sweet J. A. Bailey C. M. Munyon C. N. Luders H. O. Fastenau P. S. (2015). Visual-spatial memory may be enhanced with theta burst deep brain stimulation of the fornix: a preliminary investigation with four cases. Brain 138, 1833–1842. 10.1093/brain/awv095 26106097

[B99] Montgomery E. B. He H. (2016). Deep brain stimulation frequency-a divining rod for new and novel concepts of nervous system function and therapy. Brain Sci. 6:34. 10.3390/brainsci6030034 27548234 PMC5039463

[B100] Montgomery S. M. Betancur M. I. Buzsaki G. (2009). Behaviour-dependent coordination of multiple theta dipoles in the hippocampus. J. Neurosci. 29, 1381–1394. 10.1523/JNEUROSCI.4339-08.2009 19193885 PMC2768079

[B101] Oh Y. S. Kim H. J. Lee K. J. Kim Y. I. Lim S. C. Shon Y. M. (2012). Cognitive improvement after long-term electrical stimulation of bilateral anterior thalamic nucleus in refractory epilepsy patients. Seizure 21, 183–187. 10.1016/j.seizure.2011.12.003 22209542

[B102] Pendyam S. Bravo-Rivera C. Burgos-Robles A. Sotres-Bayon F. Quirk G. J. Nair S. S. (2013). Fear signaling in the prelimbic-amygdala circuit: a computational modeling and recording study. J. Neurophysiol. 110, 844–861. 10.1152/jn.00961.2012 23699055 PMC3742978

[B103] Prager E. M. Bergstrom H. C. Wynn G. H. Braga M. F. (2016). The basolateral amygdala γ-aminobutyric acidergic system in health and disease. J. Neurosci. Res. 94, 548–567. 10.1002/jnr.23690 26586374 PMC4837071

[B104] Rezai A. R. Sederberg P. B. Bogner J. Nielson D. M. Zhang J. Mysiw W. J.. (2016). Improved function after deep brain stimulation for chronic, severe traumatic brain injury. Neurosurgery 79, 204–211. 10.1227/NEU.0000000000001190 26702839

[B105] Roux L. Buzsáki G. (2015). Tasks for inhibitory interneurons in intact brain circuits. Neuropharmacology 88, 10–23. 10.1016/j.neuropharm.2014.09.011 25239808 PMC4254329

[B106] Rozov A. V. Valiullina F. F. Bolshakov A. P. (2017). Mechanisms of long-term plasticity of hippocampal GABAergic synapses. Biochemistry 82, 257–263. 10.1134/S0006297917030038 28320266

[B107] Saenger V. M. Kahan J. Foltynie T. Friston K. Aziz T. Z. Green A. L.. (2017). Uncovering the underlying mechanisms and whole-brain dynamics of deep brain stimulation for Parkinson's disease. Sci. Rep. 7:9882. 10.1038/s41598-017-10003-y 28851996 PMC5574998

[B108] Sanjay M. Krothapalli S. B. (2019). Modelling epileptic activity in hippocampal CA3, in Hippocampal Microcircuits: A Computational Modeller's Resource Book, 2nd edn, eds Cutsuridis C. Graham B. P. Cobb S. Vida I. (Cham: Springer-Nature Switzerland), 755–775. 10.1007/978-3-319-99103-0_26

[B109] Sankar T. Chakravarty M. M. Bescos A. Lara M. Obuchi T. Laxton A. W.. (2015). Deep brain stimulation influences brain structure in Alzheimer's disease. Brain Stimulat. 8, 645–654. 10.1016/j.brs.2014.11.020 25814404 PMC5659851

[B110] Schmidt C. van Rienen U. (2012a). Modeling the field distribution in deep brainstimulation: the influence of anisotropy of brain tissue. IEEE Trans. Biomed.Eng. 59, 1583–1592. 10.1109/TBME.2012.2189885 22410323

[B111] Schmidt C. van Rienen U. (2012b). Sensitivity analysis of the field distribution indeep brain stimulation with respect to the anisotropic conductivity of braintissue. Biomed. Tech. 57 (Suppl. 1), 4266. 10.1515/bmt-2012-4266 23096308

[B112] Schneider C. J. Bezaire M. Soltesz I. (2012). Toward a full-scale computational model of the rat dentate gyrus. Front. Neural Circ. 6:83. 10.3389/fncir.2012.00083 23162433 PMC3499761

[B113] Senova S. Chaillet A. Lozano A. M. (2018). Fornical closed-loop stimulation for Alzheimer's disease. TINS 41, 418–428. 10.1016/j.tins.2018.03.015 29735372

[B114] Siegle J. H. Wilson M. A. (2014). Enhancement of encoding and retrieval functions through theta phase-specific manipulation of hippocampus. Elife 3:e03061. 10.7554/eLife.03061 25073927 PMC4384761

[B115] Sjögren M. J. Hellström P. T. Jonsson M. A. Runnerstam M. Silander H. C. Ben-Menachem E. (2002). Cognition-enhancing effect of vagus nerve stimulation in patients with Alzheimer's disease: a pilot study. J. Clin. Psychiatry 63, 972–980. 10.4088/JCP.v63n1103 12444809

[B116] Solé-Padullés C. Bartrés-Faz D. Junqué C. Clemente I. C. Molinuevo J. L. Bargalló N.. (2006). Repetitive transcranial magnetic stimulation effects on brain function and cognition among elders with memory dysfunction. A randomized sham-controlled study. Cereb. Cortex 16, 1487–1493. 10.1093/cercor/bhj083 16339086

[B117] Somogyi P. Katona L. Klausberger T. Lasztóczi B. Viney T. J. (2013). Temporal redistribution of inhibition over neuronal subcellular domains underlies state-dependent rhythmic change of excitability in the hippocampus. Philos. Trans. R. Soc. Lond. B Biol. Sci. 369:20120518. 10.1098/rstb.2012.0518 24366131 PMC3866441

[B118] Song D. Chan R. H. Marmarelis V. Z. Hampson R. E. Deadwyler S. A. Berger T. W. (2009). Nonlinear modeling of neural population dynamics for hippocampal prostheses. Neural Netw. 22, 1340–1351. 10.1016/j.neunet.2009.05.004 19501484 PMC2821165

[B119] Song D. Robinson B.S Granacki J. J. Berger T. W. (2014). Implementing spiking neuron model and spike timing-dependent plasticity with generalized Laguerre-Volterra models. Conf. Proc. IEEE Eng. Med. Biol. Soc. 714–717. 10.1109/EMBC.2014.6943690 25570058

[B120] Sprekeler H. (2017). Functional consequences of inhibitory plasticity: homeostasis, the excitation-inhibition balance and beyond. Curr. Opin. Neurobiol. 43, 198–203. 10.1016/j.conb.2017.03.014 28500933

[B121] Stone S. S. Teixeira C. M. Devito L. M. Zaslavsky K. Josselyn S. A. Lozano A. M.. (2011). Stimulation of entorhinal cortex promotes adult neurogenesis and facilitates spatial memory. J. Neurosci. 31, 13469–13484. 10.1523/JNEUROSCI.3100-11.2011 21940440 PMC6623309

[B122] Suthana N. Aghajan Z. M. Mankin E. A. Lin A. (2018). Reporting guidelines and issues to consider for using intracranial brain stimulation in studies of human declarative memory. Front. Neurosci. 12:905. 10.3389/fnins.2018.00905 30564089 PMC6288473

[B123] Suthana N. Fried I. (2014). Deep brain stimulation for enhancement of learning and memory. Neuroimage 85, 996–1002. 10.1016/j.neuroimage.2013.07.066 23921099 PMC4445933

[B124] Suthana N. Haneef Z. Stern J. Mukamel R. Behnke E. Knowlton B.. (2012). Memory enhancement and deep-brain stimulation of the entorhinal area. N. Engl. J. Med. 366, 502–510. 10.1056/NEJMoa1107212 22316444 PMC3447081

[B125] Sweet J. A. Eakin K. C. Munyon C. N. Miller J. P. (2014). Improved learning and memory with theta-burst stimulation of the fornix in rat model of traumatic brain injury. Hippocampus 24, 1592–1600. 10.1002/hipo.22338 25087862

[B126] Titiz A. S. Hill M. R. H. Mankin E. A. Aghajan Z. Elisahi V, D. Tchemodanov N.. (2017). Theta-burst microstimulation in the human entorhinal area improves memory specificity. eLife 6:e29515. 10.7554/eLife.29515 29063831 PMC5655155

[B127] Tremblay R. Lee S. Rudy B. (2016). GABAergic interneurons in the neocortex: from cellular properties to circuits. Neuron 91, 260–292. 10.1016/j.neuron.2016.06.033 27477017 PMC4980915

[B128] Vargova G. Vogels T. Kostecka Z. Hromadka T. (2018). Inhibitory interneurons in Alzheimer's disease. Bratisl Lek Listy. 119, 205–209. 10.4149/BLL_2018_038 29663817

[B129] Vasques X. Cif L. Hess O. Gavarini S. Mennessier G. Coubes P. (2009). Stereotactic model of the electrical distribution within the internal globuspallidus during deep brain stimulation. J. Comput. Neurosci. 26, 109–118. 10.1007/s10827-008-0101-y 18553218

[B130] Vassanelli S. (2018). Implantable neural interfaces, in Living Machines: A Handbook of Research in Biomimetics and Biohybrid Systems, eds Prescott T. J. Lepora N. Verschure P. F. M. J. (Oxford University Press). 10.1093/oso/9780199674923.003.0050

[B131] Vassanelli S. Mahmud M. Girardi S. Maschietto M. (2012). On the way to large-scale and high-resolution brain-chip interfacing. Cogn. Comput. 4, 71–81. 10.1007/s12559-011-9121-4

[B132] Velasco A. L. Velasco F. Velasco M. Trejo D. Castro G. Carrillo-Ruiz J. D. (2007). Electrical stimulation of the hippocampal epileptic foci for seizure control: a double-blind, long-term follow-up study. Epilepsia 48, 1895–1903. 10.1111/j.1528-1167.2007.01181.x 17634064

[B133] Villette V. Dutar P. (2017). GABAergic microcircuits in Alzheimer's Disease models. Curr. Alzheimer Res. 14, 30–39. 10.2174/1567205013666160819125757 27539596

[B134] Wagle Shukla A. Okun M. S. (2012). Personalized medicine in deep brain stimulation through utilization of neural oscillations. Neurology 78, 1900–1901. 10.1212/WNL.0b013e318259e2af 22592364

[B135] Wang H. X. Gerkin R. C. Nauen D. W. Bi G. Q. (2005). Coactivation and timing-dependent integration of synaptic potentiation and depression. Nat. Neurosci. 8, 187–193. 10.1038/nn1387 15657596

[B136] Wei X. F. Grill W. M. (2005). Current density distributions, field distributions andimpedance analysis of segmented deep brain stimulation electrodes. J. Neural Eng. 2, 139–147. 10.1088/1741-2560/2/4/010 16317238

[B137] Wester J. C. McBain C. J. (2014). Behavioral state-dependent modulation of distinct interneuron subtypes and consequences for circuit function. Curr. Opin. Neurobiol. 29, 118–125. 10.1016/j.conb.2014.07.007 25058112 PMC4268408

[B138] Witter M. (2019). Connectivity of the hippocampus, in Hippocampal Microcircuits: A Computational Modeler's Resource Book, 2nd edn, eds Cutsuridis V. Graham B. P. Cobb S. Vida I. (Cham: Springer-Nature Switzerland).

[B139] Yu G. J. Hendrickson P. J. Song D. Berger T. W. (2019). Spatiotemporal patterns of granule cell activity revealed by a large-scale, biologically realistic model of the hippocampal dentate gyrus, in Hippocampal Microcircuits: A Computational Modeller's Resource Book, 2nd edn, eds Cutsuridis C. Graham B. P. Cobb S. Vida I. (Cham: Springer-Nature Switzerland), 473–508.

[B140] Zhang C. Hu W. H. Wu D. L. Zhang K. Zhang J. G. (2015). Behavioral effects of deep brain stimulation of the anterior nucleus of thalamus, entorhinal cortex and fornix in a rat model of Alzheimer's disease. Chin. Med. J. 128, 1190–1195. 10.4103/0366-6999.156114 25947402 PMC4831546

